# Bacterial methyltransferases: from targeting bacterial genomes to host epigenetics

**DOI:** 10.1093/femsml/uqac014

**Published:** 2022-08-10

**Authors:** Monica Rolando, Cristina Di Silvestre, Laura Gomez-Valero, Carmen Buchrieser

**Affiliations:** Institut Pasteur, Université de Paris, CNRS UMR 6047, Unité Biologie des Bactéries Intracellulaires, 28, Rue du Dr. Roux, 75724 Paris Cedex 15, France; Institut Pasteur, Université de Paris, CNRS UMR 6047, Unité Biologie des Bactéries Intracellulaires, 28, Rue du Dr. Roux, 75724 Paris Cedex 15, France; Institut Pasteur, Université de Paris, CNRS UMR 6047, Unité Biologie des Bactéries Intracellulaires, 28, Rue du Dr. Roux, 75724 Paris Cedex 15, France; Institut Pasteur, Université de Paris, CNRS UMR 6047, Unité Biologie des Bactéries Intracellulaires, 28, Rue du Dr. Roux, 75724 Paris Cedex 15, France

**Keywords:** methyltransferase, epigenetics, bacterial pathogens, *Legionella*

## Abstract

Methyltransferase (MTases) enzymes transfer methyl groups particularly on proteins and nucleotides, thereby participating in controlling the epigenetic information in both prokaryotes and eukaryotes. The concept of epigenetic regulation by DNA methylation has been extensively described for eukaryotes. However, recent studies have extended this concept to bacteria showing that DNA methylation can also exert epigenetic control on bacterial phenotypes. Indeed, the addition of epigenetic information to nucleotide sequences confers adaptive traits including virulence-related characteristics to bacterial cells. In eukaryotes, an additional layer of epigenetic regulation is obtained by post-translational modifications of histone proteins. Interestingly, in the last decades it was shown that bacterial MTases, besides playing an important role in epigenetic regulations at the microbe level by exerting an epigenetic control on their own gene expression, are also important players in host–microbe interactions. Indeed, secreted nucleomodulins, bacterial effectors that target the nucleus of infected cells, have been shown to directly modify the epigenetic landscape of the host. A subclass of nucleomodulins encodes MTase activities, targeting both host DNA and histone proteins, leading to important transcriptional changes in the host cell. In this review, we will focus on lysine and arginine MTases of bacteria and their hosts. The identification and characterization of these enzymes will help to fight bacterial pathogens as they may emerge as promising targets for the development of novel epigenetic inhibitors in both bacteria and the host cells they infect.

## Teaser

Bacterial methyltransferases play a role in epigenetic regulations in bacteria as well as in controlling the host epigenetic landscape during infection

## Introduction

**Figure 1.**
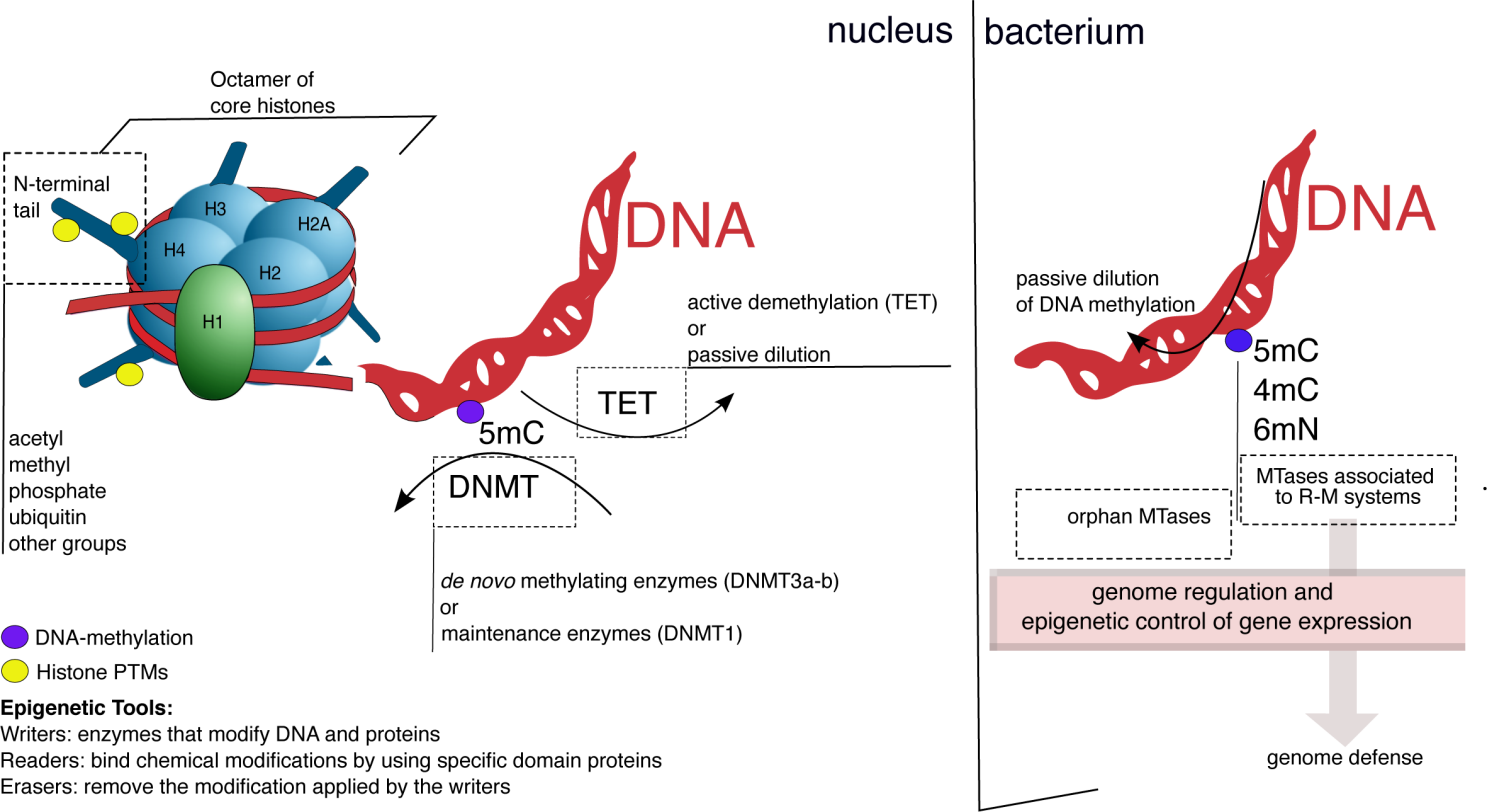
The epigenomes of eukaryotes and bacteria. In eukaryotes, epigenetic modifications involve DNA methylation (purple tag) and histone modifications [yellow tag; for a complete list of possible chemical modifications on histone proteins please refer to Huang et al. (2014) and Zhao and Garcia (2015)]. The DNA is packaged in a structure called chromatin, which regulates its activity and inheritance and is organized in fundamental structures called nucleosomes. Each nucleosome consists of a segment of 147 bp of DNA wrapped around an octamer of proteins containing two copies each of four different histones: H2A, H2B, H3, and H4. This arrangement of 11 nm of DNA and its associated proteins forms a fiber, which plays a major role in the cell. In fact, the regulatory proteins that interact with target subunits of the fiber can increase or decrease the compactness of the chromatin structure, leading to enhancing or reducing gene expression. Separate enzymes are responsible for *de novo* methylation and the maintenance of DNA methylation. DNA demethylation can occur by an active process driven by dedicated proteins (TET enzymes), or by a passive one, where DNA methylation is diluted upon DNA replication. Typically, the methylated base in eukaryotes is C5m, whereas it is often N6m in bacteria. Most bacterial DNA-methyltransferases (MTases) belong to the restriction–modification (R–M) system, responsible for genome defense, whereas orphan DNA MTases have no apparent cognate REase. Both kinds of MTases play a role in epigenetic regulations in bacteria.

The epigenome consists of a network of modifications on nucleotides or, in the case of eukaryotes, on histone proteins, that lead to the alteration of the biochemical landscape of DNA, without altering the DNA sequence, but directly impacting its structural conformation and, therefore, affecting transcriptional regulation. In eukaryotes, epigenetic regulation involves DNA methylation and histone post-translational modifications, whereas in bacteria, which lack histone proteins, epigenetic control relies on DNA methylation only (Fig. 1).

The plethora of enzymes catalyzing these modifications on DNA and histones are categorized in three functional classes: the writers and erasers, a dedicated group of enzymes that add and remove, respectively, various chemical modifications; and the readers, specialized domain containing proteins that identify and interpret those modifications (Venkatesh and Workman 2015). Epigenetic signatures and their functional consequences contribute to the normal development of an organism, but also environmental factors influence the epigenetic state, and consequent regulation. In addition to many studies into these diverse regulations in eukaryotic cells, it was shown how some bacterial pathogens can influence and in a certain way govern the epigenetic state of host cells in a dynamic manner by directly modifying the chromatin.

In this review, we discuss a specific class of epigenetic writers, methyltransferases (MTases), that methylate both DNA and proteins, thereby acting as epigenetic tools, from bacteria to eukaryotes.

## MTases: an eclectic class of enzymes

MTases are a large group of enzymes that, for their majority, methylate their substrate using S-adenosyl-L-methionine (AdoMet or SAM) as methyl donor. The methyl group may be transferred to form methylated derivatives of proteins, lipids, polysaccharides, nucleic acids, and various small molecules. Methylation reactions are essential transformations in biology, as they control countless cellular processes by transforming the metabolism of molecules. Here, we will focus on methylation of nucleotides (DNA or RNA) and proteins as central components of the epigenetic machinery of both prokaryotic and eukaryotic organisms.

### DNA-methylation: setting up the epigenome

DNA-MTases catalyze the transfer of a methyl group from SAM to cytosine or adenine bases embedded in a specific DNA sequence. The methyl group is positioned in the major grove of the DNA helix, where it can easily attract or repel various DNA-binding proteins. Such a methylation adds postreplicative extra information to the DNA without changing the original sequence, as newly synthetized DNA strands do not carry any methylation (Jeltsch 2002). The C-5 and N-4 positions of cytosine and N-6 position of adenine are the target sites for methylation. All three methylation patterns are found in prokaryotes, but it was thought that in eukaryotes only the methylation of cytosine at the C-5 position exists. However, a recent in-depth analysis of rarely modified bases demonstrated that N-6 adenine can also be methylated in eukaryotes (Wu et al. 2016, Zhu et al. 2018).

Prokaryotic DNA-MTases can be classed in two major groups, depending on the position of the base they target in the double helix. Endocyclic MTases target the cytosine C-5, and exocyclic amino MTases methylate the adenine at the N-6 position or the cytosine at N-4 position (Bheemanaik et al. 2006). In general, all DNA MTases show structural similarity, in particular the SAM-binding domain is well conserved across kingdoms. In contrast, the target recognition domain shows high variability in sequence and structure and is closely tied to the target specificities (Malone et al. 1995). In mammalian cells, DNA methylation patterns are established during embryonic development by *de novo* methylating enzymes called Dnmt3a and Dnmt3a, and maintained by a Dnmt1-mediated copying mechanism during cell division (Jones and Liang 2009).

DNA methylation marks are chemically stable. In bacteria the removal of the methylation is usually achieved by two rounds of DNA replication (passive demethylation), in contrast in eukaryotes DNA methylation can also be actively removed. Passive demethylation simply requires the impairment of the maintenance of the DNA methylation machinery, which results in a 2-fold dilution of methyl-CpG groups during each round of DNA synthesis, whereas active demethylation occurs via the action of the Ten–eleven translocate (TET) family of dioxygenases, through a complex cycle of repeated oxidations (He et al.^2011^, Ito et al. 2011). TET-like genes have also been identified in bacteria; thus it is likely that such systems might also function in prokaryotes (Iyer et al. 2009).

In bacteria and eukaryotes, DNA-methylation is generally associated with transcriptional repression. The methyl group of 6mA,  5mC, and 4mC protrudes from the major groove of the double helix, thereby providing a platform for DNA-binding proteins to bind cognate nucleotide sequences. 5-cytosine methylation (5mC) is typically involved in the control of eukaryotic transcription and is associated with gene silencing. This modification is conserved across all kingdoms of eukaryotes, where it is generally found in the CpG dinucleotide context, where the cytosine in the dinucleotide sequence 5’-CpG-3’ is modified. Its best characterized function is the repression of the transcription of potentially deleterious transposable elements (TEs), however, it also plays an important role in silencing of germline-specific genes and in developmental processes, such as X-chromosome inactivation via transcriptional silencing (Jones 2012). Indeed, transcription can be regulated by controlling DNA-methylation of specific regions found mainly directly upstream of gene promoters, containing clusters of CpG sequences that are named CpG islands (Deaton and Bird 2011). Methylation of these regions leads to a drastic gene repression by interfering with transcription factors (Zhu et al. 2016), as well as by the direct binding of a family of proteins, known as methyl-CpG binding domain proteins (MBDs). MBDs bind DNA-methylated CpGs and recruit repressor complexes to methylated promoter regions, thereby contributing to transcriptional silencing (Du et al. 2015). Transcriptional activation is regulated via multiple mechanisms involved in protecting CpG islands from *de novo* methylation and thereby maintaining these regions unmethylated (Weber et al. 2007)

In bacteria, DNA-methylation is often found at N-6 adenine (6mA) and N-4 cytosine (4mC). In particular, it has been established a clear link between 6mA and transcriptional regulation of essential processes such as conjugation, regulation of DNA replication initiation, cell cycle control, nucleoid reorganization, DNA mismatch repair, transcriptional regulation of housekeeping and virulence genes, and post-transcriptional gene regulation [see Chapter 2/and Wion and Casadesús (2006)].

### RNA-methylation as a part of the epitranscriptome

In the last decade, a new field of research, RNA modifications, added an additional layer of complexity to gene regulation. Indeed, similar to DNA, cellular RNAs and especially the so-called regulatory RNAs—including miRNAs, piRNAs, endogenous siRNAs, and long noncoding RNAs—may be decorated with diverse chemical modifications (Roundtree et al. 2017). If RNAs were once thought of as a gene expression intermediate only, today it is well-established that RNA modifications provide an additional layer of gene-expression control. This emerging field of investigations is referred to as “RNA epigenetics,” or “epitranscriptomics” (Saletore et al. 2012).

RNAs from all kingdoms of life can be post-transcriptionally modified with more than 150 chemically distinct additions known to date (Boccaletto et al. 2018), that influence RNA folding and function. Methylation is the most common RNA modification as roughly two-thirds of RNA modifications involve the addition of methyl groups. In particular, m6A RNAs are found in all kingdoms of life.

In eukaryotes, 57 RNA MTases have been identified, targeting different bases and riboses of coding and noncoding RNAs [for a complete review on human RNA MTase classification and their targets, see Schapira (2016) and Romano et al. (2018)]. Among them, N6-Methyladenosine (m6A), first observed more than 40 years ago (Desrosiers et al. 1974), is the most abundant mark on eukaryotic mRNAs (one-third of transcripts) and ncRNAs and represents one of the best-studied RNA modifications so far (He and He 2021, Zaccara et al. 2019). Further interest in m6A mRNA has recently emerged, due to the identification of specific demethylases such as FTO and ALKB5H, both belonging to the AlkB family of dioxygenases responsible for converting m6A to adenosine (Jia et al. 2011, Zheng et al. 2013). These findings, together with the identification of m6A readers (Wang et al. 2014), support the idea that chemical modifications on RNAs could represent a reversible and dynamic mode of post-transcriptional regulation (Shi et al. 2019). In fact, the dynamic equilibrium between methylated and unmodified RNA bases, including m6A but also other methylated bases (Wiener and Schwartz 2021), controls a variety of physiological relevant processes, such as splicing, stability, turnover, nuclear export, and mediation of cap-independent translation, showing that it is a dynamic process.

In bacteria, methylated adenosines play a structural role in increasing the efficiency of stacking in ncRNAs, such as ribosomal RNAs. An example is *Escherichia coli*, that contains two m6A residues on its 23S rRNA: these methylated adenosines have been shown to protrude from the RNA loop and form stacking interactions within the ribosomal RNA (Kierzek and Kierzek 2003). However, mechanisms that connect methylation of bacterial RNAs with gene expression are still unknown, as in bacteria, the majority of methylations, and chemical modifications in general, are located in tRNAs and rRNAs (Boccaletto et al. 2018). Likewise, the work of Deng et al. (2015) demonstrated that m6A is also an abundant mRNA modification in *E. coli* and *Pseudomonas aeruginosa* as high-resolution transcriptome wide m6A profiling revealed a conserved and distinct m6A distribution pattern.

### Protein MTases modifying chromatin

Nitrogen (N-) or oxygen (O-) atoms are the most common targets for protein methylation. The amino acids targeted by N-methylation are lysine, arginine, histidine, glutamine, and asparagine, whereas O-methylation targets the carboxyl groups of glutamate and aspartate. Protein methylation, like other chemical modifications on amino acids, influences the local charge of the molecule, by increasing its hydrophobicity. Also, methylation of negatively charged amino acids can significantly affect their 3D shape, and consequently their function.

In eukaryotes, protein MTases control epigenetic regulation by targeting histone proteins, specifically lysine and arginine methylations of histones have been first described in the late 1960s and have been extensively characterized since then (Murn and Shi 2017). DNA is wrapped around histone proteins to form a complex called chromatin that allow the DNA to be packaged up and condensed. Dynamic histone modifications may, therefore, control DNA packaging and regulate nuclear regulation. In total, two main classes of histone protein MTases, the enzymes encoding a SET [Su(var), E(z), and Trithorax] domain and those encoding a Rossmann fold, also known as 7-β-sheets family, have been described to date (Falnes et al. 2016, Gana et al. 2013). In human cells, SET domain containing MTases constitute a family of about fifty proteins acting as lysine MTases (PKMTs) that methylate various N-terminal lysine residues of histones H3 and H4 (Dillon et al. 2005). Proteins belonging to the Rossmann fold family are arginine MTases (PRMTs), comprising nine enzymes (designated PRMT1-9), and the PKMT named DOT1L (disruptor of telomeric silencing), a structurally unique enzyme that has been shown to overlay with arginine MTases, rather than SET-domain containing enzymes (Min et al. 2003). DOT1 L exclusively methylates Ly79 in the globular region of histone H3 (H3K79), leading to sequential mono-, di- and trimethylated forms (Feng et al. 2002). Lysine can exist in four methylation states (unmodified, mono- di-, or trimethylated), whereas arginine can be unmodified, mono- or dimethylated (dimethylated arginine residues can occur in either symmetric—two separate nitrogen atoms—or asymmetric—same nitrogen). For a complete review on enzymatic properties of lysine and arginine MTases, please refer to Boriack-Sjodin and Swinger (2016). As previously mentioned for DNA MTases, histone protein methylations, together with other PTMs on histone proteins, alter noncovalent contacts within and between nucleosomes, thereby impacting on their function. Considering the substantial number of lysines that can be methylated, each with multiple methylation states, histone modifications regulate an array of biological processes. Notably, the location and the degree of methylation of a particular residue is associated with a particular transcriptional state or chromatin structure, as it is now generally accepted that histone modifications serve as signals for the recognition by effectors or reader proteins, which impact chromatin structure and function (Lee et al. 2010).

The dynamic status of these modifications implies the existence of histone demethylases. Indeed, histone lysine demethylases (KDMs) remove methyl group(s) from lysines, and arginine demethylases (RDMs) from arginines. A total of eight subfamilies of histone lysine demethylases (KDM 1–8) have been characterized and classed in two major groups: (i) Lys-specific demetylases or LSD demethylases, which were the first reported KDMs, that oxidize the ε-amino group of Lys, thus they allow only demethylation of mono- and dimethyl lysines; and (ii) α-ketoglutarate-dependent Jumonji C-terminal domain (JMJC)-containing demethylases, that oxidize the attached methyl group, which allow the demethylation of mono-, di-, and trimethyl lysines (Kooistra and Helin 2012). In contrast, arginine demethylases (RDMs), are not very well-characterized although several studies indicated the reversibility of this modification. To date, two histone RDMs have been reported, PAD4 (Wang et al. 2004) and JMJD6 (Chang et al. 2007), however, their activities have been questioned meanwhile. Furthermore, several KDMs have been shown to also demethylate arginines (Walport et al. 2016).

While epigenetic regulation by DNA methylation in bacteria is increasingly studied, how protein modifications may impact DNA processes remain an open question. In bacteria, DNA interacts with small, basic, nucleoid-associated proteins (NAPs), which are responsible for chromosome compaction and the coordination of DNA replication and transcription (Dillon and Dorman 2010). Although some of them have been referred to as “histone-like proteins” (HUs) and have been shown to function as transcriptional coactivators and corepressors (Aki and Adhya 1997), the possibility that bacteria specifically modulate their chromatin proteins by PTMs is still under discussion (Carabetta 2021).

### 2/Epigenetics in bacteria: main role of DNA-MTases

Most bacterial DNA-MTases belong to restriction–modification (R–M) systems, that were first recognized in *E. coli* for limiting and regulating bacteriophage infections (Gold et al. 1963). R–M systems are ubiquitous in the bacterial world and generally encode two enzymes with the same DNA binding specificity: a DNA adenine MTase, that modifies a specific target sequence in the host genome to protect it from cleavage of a target sequence-specific endonuclease (REase), that cleaves unmethylated or inappropriately methylated targets from exogenous DNA. They are typically regarded as innate defense systems as they serve to identify and eliminate foreign DNA providing a barrier against genetic flux between different lineages (Mruk and Kobayashi 2014). R–M systems are classified in four major types, differing in their molecular structure, sequence recognition, cleavage position, and cofactor requirements (Roberts et al. 2003).

Oliveira and Fang (2020) recently reported a total of 26582 MTases in 5568 complete bacterial genomes, with Type II MTases present in the highest density. Moreover, 52% of the species harbor persistent MTases, defined as conserved in at least ≥ 80% of the genomes of each species, that recognize the same target sites on DNA (Oliveira and Fang 2020). Some DNA MTases, known as orphans, have no apparent cognate REase gene and, although a possible origin from degraded R–M systems has been hypothesized, it seems clear that the majority are acquired by horizontal gene transfer in their orphan state and further kept due to strong selective pressure (Oliveira et al. 2014). Often, orphan MTases are involved in genome regulation and epigenetic control of gene expression as, in contrast to methylation by R–M systems, methylation by orphan MTases (whether persistent or not) often produces patters of DNA methylation that are consistent with gene regulatory functions (Blow et al. 2016, Oliveira and Fang 2020). Interestingly, Blow et al. (2016) showed that, if MTases of R–M systems are almost always associated with complete DNA modifications of their genomes, consistent with their role in protecting the genome from the cognate restriction enzymes, the orphan MTases are associated with small subsets of consistently unmethylated sites throughout the genome. This distinctive signature of orphan MTases represents a regulatory mechanism of gene expression, in fact the majority of orphan MTases are associated with a substantial enrichment of unmethylated motifs in regulatory regions of the genome (Blow et al. 2016). An example for an orphan and persistent MTase is the deoxyadenosine MTase Dam, i.e. widespread in γ-proteobacteria. It catalyzes postreplicative formation of 6mA in the palindromic 5’-GATC-3’ motif (Brooks et al. 1983), and controls transcription of specific genes that regulate diverse processes, including DNA replication timing, DNA repair, nucleoid segregation, phase-variation switches, but also virulence of bacterial pathogens (Adhikari and Curtis 2016, Marinus and Casadesus 2009). For instance, in *Salmonella typhimurium* Dam controls bacterial virulence (Heithoff et al. 1999) and the expression of the pathogenicity island-1 (SP-1; Balbontín et al. 2006). In *Yersinia enterocolitica* Dam overproduction causes both transcriptional and post-transcriptional alterations in the synthesis of virulence factors (Fälker et al. 2007).

MTases are thus key factors for the regulation of gene expression, in particular in the context of phase variation mechanisms that control the formation of phenotypically distinct cells in populations of genetically identical bacteria. Reversible and high-frequency transitions between two distinct states, resulting in ON/OFF switching of expression, are usually mediated by mutations at genomic repeat sequences located either within the genes encoding variant proteins, or in their promoter regions, as well as by site-specific recombination or epigenetic regulation mediated by DNA-methylation (Woude 2011).

Pathogenic bacteria, that are frequently challenged by rapid changing environments such as immune defenses of their hosts and have to adapt to continuous selective pressure over many individual cycles of transmission, often regulate expression of their virulence genes by phase variation. Classical examples are bacterial surface factors required for initial adherence for host colonization, such as pili, adhesins, flagella, and lipopolysaccharide (Phillips et al. 2019a). The first characterized and most studied example is the *pap* (pyelonephritis-associated pili) operon of uropathogenic *E. coli* that encodes fimbriae for adhesion to the urinary epithelium. The expression of the operon is regulated by the formation of DNA-methylation patterns by the MTase Dam, i.e. regulating transcription factor binding in regulatory regions, thereby leading to ON/OFF switches (Blyn et al. 1990, Woude et al. 1996). If we consider that human-adapted pathogens have many phase-variable genes, the combinatorial power of this contingency strategy to generate a highly diverse population becomes apparent. For example, almost 100 putative phase-variable genes have been identified in *Neisseria* spp., indicating the huge potential for diversification mediated by gene switching during host colonization (Snyder et al. 2001).

Orphan DNA MTases were thought to be the only ones playing a role in epigenetic regulations in bacteria, and MTases associated to R–M systems were thought to function “only” in genome defense. However, an increasing number of MTases associated with R–M systems has recently been shown to have additional roles in transcriptional regulation and formation of phenotypic cell variants.

Indeed, many bacterial pathogens contain MTases associated with R–M systems that are subject to phase variation. Phase variation of the expression of DNA MTase results in differential DNA methylation patterns throughout the genome, translating into expression changes of multiple genes via epigenetic mechanisms. These systems are called phasevarions (phase-variable regulons). The concept of phasevarions was first described in *Haemophilus influenzae*, where phase-variable ON/OFF switching of a Type III DNA MTase results in the phase variation of an entire regulon that differentiates the bacterial cell into two alternative expression states with multiple phenotypic differences (Srikhanta et al. 2005). Since then, phase-variable Type III and Type I restriction–modification systems that control a regulon of genes via changes in global DNA methylation caused by the phase variation of the restriction–modification systems, have been reported in a range of human-adapted bacterial pathogens. For a complete and recent review on the identification and analysis of phase-variable expressed DNA MTases associated with R–M systems and their epigenetic regulation of virulence and immune-evasion in human-adapted bacterial pathogens please refer to (Seib et al. 2020).

Although the majority of studies focused on the activity of adenine MTases as epigenetic regulators, recent works started to explore the role of 5mC and 4mC in epigenetic signaling. The formation of 5mC may influence gene expression in *Helicobacter pylori*, via the activity of the JHP1050 MTase (Estibariz et al. 2019), in *Vibrio cholerae*, where the VchM orphan 5mC MTase has been shown to be necessary for optimal growth during infection (Chao et al. 2015), or in *E. coli* (Kahramanoglou et al. 2012, Militello et al. 2014). 4mC has recently been shown to affect gene expression and virulence related traits in *H. pylori* (Kumar et al. 2018). The recent identification of these methylations in bacterial genome suggests that DNA methylation remains still a poorly understood component of prokaryotic life and it plays a much deeper role than currently thought.

### 3/Bacterial MTases and their function in targeting the host

During bacterial infection, host cells activate a series of proinflammatory responses to avoid microbial colonization and to delay bacterial spread. Thus, pathogenic bacteria evolved a wide range of strategies to avoid eradication by their hosts. In particular, elaborate secretion systems inject virulence factors, named effectors, into host cells in order to subvert host defenses (Galán and Waksman 2018). These protein effectors allow bacteria to compete with other microorganisms colonizing the same niche and to interact with the host signaling pathways for blocking, or delaying, the host cell response and to promote their own survival. Furthermore, many of these effectors hijack the host cell response through chemical modifications by the addition or removal of functional groups to host proteins or nucleotides to destabilize the host and to overcome host defenses.

For a long time, it was believed that bacterial effectors released by the different secretion systems act only in the host cytosol (Rapisarda and Fronzes 2018). However, in late 1977 a segment of the Ti plasmid, thereafter named T-DNA of *Agrobacterium turmefaciens*, had been found integrated into plant cells infected by this phytopathogen (Hooykaas et al. 1977). Virulent *Agrobacterium* strains transfer single strand T-DNA and several virulence effector proteins (mainly VirD2) through a specialized type-4 secretion system (T4SS) into plant cells. The single stranded T-DNA traverses the host cell cytoplasm and enters the host cell nucleus, where it eventually integrates in the host genome, promoting uncontrolled cell proliferation and interfering with host transcription mechanisms to produce nutrients essential for bacterial survival (Gelvin 2017).

**Figure 2.**
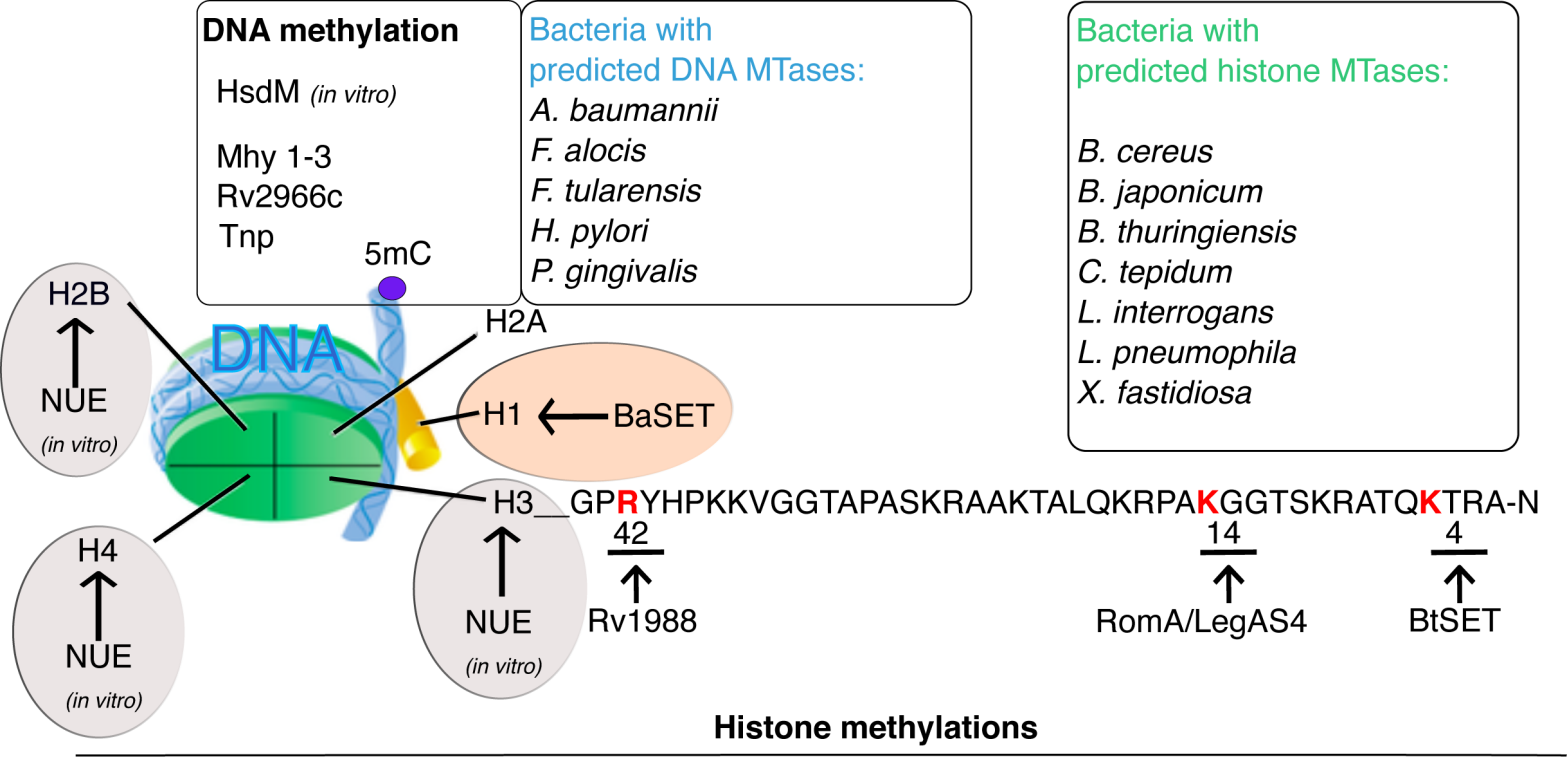
Schematic representation of identified bacterial effectors targeting the nucleus and methylating DNA or histone proteins. Top: identified bacterial DNA-MTases as well as putative effectors. Bottom: identified bacterial histone MTases as well as putative effectors.

In the last decade, a new family of bacterial, secreted effectors has been characterized, the so-called nucleomodulins (Bierne and Cossart 2012). Derived from the combination of the words “nucleus” and “modulins,” these proteins represent a group of molecules that enter the host cell nucleus to hijack nuclear functions such as modulating the expression of key genes for the host cell response to the pathogen. The last decade has witnessed an increase in the number of nucleomodulins identified, targeting various nuclear elements (Bierne and Pourpre 2020). Most of the bacterial nucleomodulins secreted in the host cell manipulate the chromatin organization of the infected cell both in direct and indirect ways, interfering with transcriptional programs necessary for the cell survival. We will discuss here the nucleomodulins that function as MTases and interact with both DNA and histone proteins, to modify the chromatin architecture and enhance bacterial colonization (Fig. 2 and Table 1).

**Table 1.** Bacterial nucleomodulins functioning as MTases in the host.

**Pathogen**	**Effector**	**Nuclear localization**	**Activity/target**	**Reference**s
DNA MTase nucleomodulins				
*Klebsiella pneumoniae*	HsdM	Yes	Methylates eukaryotic DNA *in vitro*	Lee et al. (2009)
*Mycobacterium tuberculosis*	Rv2966c	Yes	Cytosine MTase (non-CpG context)—targets promoter sequences of several interleukin receptor genes	Sharma et al. (2015)
*Mycoplasma hyorhinis*	Mhy1-3	Yes	CG- and GATC-specific MT-ases—target pro-oncogenic and proliferation genes	Chernov et al. (2015)
*Filifactor alocis*	DNMTs		Unknown	Aruni et al. (2014)
*Francisella tularensis*	DNMT		Unknown	Champion (2011)
*H. pylori*	DNMTs		Unknown	Sitaraman (2014)
*Acinetobacter baumannii*	DNMT	Yes	Unknown	Moon et al. (2012a)
Arginine and lysine MTase nucleomodulins				
*M. tuberculosis*	Rv1988	Yes	Arg-MTase—targets H3R42–induces gene repression	Yaseen et al. (2015)
*Chlamydia pneumoniae*	cpnSET	Yes	SET-domain MTase—targets H3 and Hc1	Murata et al. (2007)
*Chlamydia trachomatis*	NUE	Yes	SET-domain MTase—targets H2B/H3/H4 and automethylates	Pennini et al. (2010)
*Burkholderia pseudomallei*	BtSET	Yes	SET-domain MTase—targets H3K4 on rRNA	Li et al. (2013)
*Burkholderia thailandesis*				
*Bacillus anthracis*	BaSET	Yes	SET-domain MTase—targets H1, silencing of host inflammatory response	Mujtaba et al. (2013)
*Methanosarcina mezei* (archea)	Gö1-SET		SET-domain MTase—targets H4 *in vitro*	Manzur and Zhou (2005)
*Legionella pneumophila* strain Paris	RomA	Yes	SET-domain MTase—targets H3K14, silences host response to infection	Rolando et al. (2013)
*L. pneumophila* strain Philadelphia	LegAS4	Yes	SET-domain MTase—targets H3K4 on rDNA	Li et al. (2013)
*L. pneumophila*	LppDOT1L		Unknown	Gomez-Valero et al. (2019)
*L. pneumophila*	PRMTs		Unknown	Cazalet et al. (2004)

### Bacterial DNA MTAses targeting the host

The first bacterial DNA-MTase shown to target the host cell nucleus was HsdM of *Klebsiella pneumoniae* (Lee et al. 2009). HsdM belongs to the Type-I bacterial R–M systems (Taylor et al. 2010) and has a nuclear localization signal (NLS) that targets it to the nucleus when expressed in human cell lines. The function of HdsM is not known, but it was shown that recombinant HsdM methylates eukaryotic DNA *in vitro* (Lee et al. 2009).

Another secreted bacterial MTase is Rv2966c, encoded by *Mycobacterium tuberculosis* that targets cytosines in a non-CpG dinucleotide context (Sharma et al. 2015)*. Mycobacterium tuberculosis* is an intracellular pathogen responsible for human tuberculosis, colonizing the human lung via inhalation of bacteria-containing droplets. Despite pressure from innate immune cells in the lungs, it can persist in this environment (Bussi and Gutierrez 2019). Rv2966c is enhancing the persistence of the pathogen in the lungs by methylating specific DNA sequences generating hypermethylated regions in promoter sequences of several interleukin receptor genes. Interestingly, Rv2966c, like the mammalian DNA MTase DNMT3L, can also interact with histones H3 and H4, supporting the idea that this bacterial effector interacts with the host epigenetic machinery at multiple levels (Sharma et al. 2015).

The second identified family of nucleomodulins that selectively and efficiently methylate host cell DNA are the secreted effectors Mhy1-3 encoded by *Mycoplasma hyorhinis* (Chernov et al. 2015). *Mycoplasma* are parasitic microbes that in humans frequently populate mucosal surfaces and persist as long-term asymptomatic infections, likely promoting chronic aberrant states in infected tissues, often terminating in tumors (Benedetti et al. 2020). Chernov et al. (2015) demonstrated that three *M. hyorhinis* CG- and GATC-specific MT-ases efficiently translocate to the host cell nucleus leading to a high degree of methylation of the human genome, and thereby stimulating pro-oncogenic and proliferation pathways in human cells. Given that this pathogen is generally associated with prostate and gastric cancer, the authors hypothesize that either the infection contributes to the malignancy onset or, alternatively, that tumors provide a favorable environment for mycoplasma growth that may facilitate further dissemination (Chernov et al. 2015).

Other pathogens have been reported to code for DNMTs in their genomes, however, no functional analyses of those putative nucleomodulins have yet been performed. Examples are, *Porphyromonas gingivalis* and *Filifactor alocis*, major pathogens associated with periodontal disease, with as many as 18 different methyltransfeases predicted in the genome of *F. alocis* (Aruni et al. 2014). Although chronic infection by *P. gingivalis* has been shown to introduce *de novo* DNA methylation at several CpGs located in the TLR2 promotor region (Benakanakere et al. 2015), a direct role of these bacterial MTases in host chromatin modifications and epigenetic changes awaits confirmation. Similarly, *Francisella tularensis*, a human intracellular pathogen, encodes a MTase likely mimicking eukaryotic MTase of its hosts (Champion 2011). A high number of DNMTs has also been predicted in the genome of *H. pylori*, a Gram-negative, microaerophilic, and spiral-shaped bacterium, i.e. associated with upto 10% of patients that develop duodenal ulcer disease (Suerbaum and Michetti 2002). *Helicobacter pylori* infection induces CpG methylation in the promoter region of mismatch repair and tumor suppressor genes, which are associated with the initiation and progression of gastric cancer (Kaise et al. 2008). Although it was predicted in the different strains sequenced that *H. pylori* encodes 25 to 37 adenine- and cytosine-specific MTases (Sitaraman 2014, Vitkute et al. 2001), their functional role during infection remains to be investigated.

A DNA–cytosine MTase has also been annotated in the genome of *Acinetobacter baumannii*, an important opportunistic pathogen that causes a variety of human infections, with high mortality rate in immunocompromised patients (Antunes et al. 2011). Cytotoxic effects have been shown when this nucleomodulin is translocated into the host cell nucleus (Moon et al. 2012a), however, direct DNA methylation has not been investigated. In contrast, another nuclear effector of *A. baumannii*, a transposase (Tnp) delivered to host cells via outer membrane vesicles localizes in the nucleus where it induces DNA methylation in the CpG regions of the gene coding for E-cadherin, inducing its transcriptional down-regulation (Moon et al. 2012b). The molecular mechanism describing how Tnp induces DNA methylation and its possible interaction with the nuclear localized DNMT remains unclear.

### Bacterial lysine and arginine MTases: a subclass of nucleomodulins targeting host histones

As described above, histone methylation mostly occurs on arginine and lysine residues. Several nucleomodulins that directly methylate histone proteins have been characterized: until now, only one nucleomodulin that encodes PRMT activity has been identified, but several that target lysines via SET-domains.

Rv1988, secreted by *M. tuberculosis* is an arginine MTase that targets the host chromatin where it dimethylates, instead of “classical” target residues located in the histone tails, a noncanonical arginine residue (R42) located in the core region of histone H3 (Yaseen et al. 2015). Hence, H3R42 is located at critically important entry/exit points of DNA in the nucleosome that have the potential to change nucleosomal dynamics and affect transcription (Casadio et al. 2013). Indeed, Rv1988-driven H3R42 dimethylation induces a repression of genes that are important for the immune response against mycobacterial infection (Yaseen et al. 2015).

The first bacterial, nuclear effector encoding a SET-domain MTase, has been described in *Chlamydia pneumoniae* (Murata et al. 2007). Chlamydiae exhibit a unique life cycle by alternating between an infectious but transcriptionally inactive, elementary body (EB) that enters the host cell, and a noninfectious reticulate body (RB), i.e. transcriptionally active and replicates intracellularly. Genome analyses revealed genes predicted to encode a SET-domain containing protein (Stephens et al. 1998). Murata et al. (2007) showed that cpnSET is a nucleomodulin that methylates lysine residues of murine histone H3 *in vitro* and interacts with and methylates the histone H1-like protein Hc1of *C. pneumoniae*. It is suggested that the chlamydial histone-like proteins Hc1 and Hc2 may act as global transcriptional regulators and play a role in compacting DNA during the RB to EB transition (Perara et al. 1992). These findings suggested that the cpnSET effector may play an important role in the morphological changes from RBs to EBs, but it may also be involved in the modification of the chromatin folding of the host. The secretion of this SET-domain containing chlamydial effector was proven by Pennini et al. (2010), when they demonstrated that the homologue of cpnSET in *C. trachomatis*, named NUE, is translocated in the cytosol by a type-3 secretion system (T3SS), where it translocates to the nucleus of infected cells (Pennini et al. 2010). Moreover, they showed that NUE methylates lysines of histones H2B, H3, and H4 *in vitro*, and is capable of automethylation, a characteristic that might play a role in enhancing histones MTase activity.

Another functional SET-domain MTase is encoded by *Burkholderia* spp. (Li et al. 2013). BtSET is a nucleomodulin secreted by pathogenic *Burkholderia pseudomallei* and nonpathogenic *Burkholderia thailandesis* through a specialized T3SS. This enzyme targets histone H3 at lysine 4 (H3K4) for mono- and dimethylation of rRNA, thereby increasing transcription of rRNA genes. A mechanism that seems to facilitae *Burkholderia* multiplication in host cells.

A SET domain containing protein is also encoded by the Gram-positive bacterium *Bacillus anthracis*, the etiological agent of anthrax, a zoonotic disease that can be lethal for humans (Moayeri et al. 2015). *Bacillus anthracis* secretes a nucleomodulin named BaSET, that localizes in the nucleus of infected cells where it specifically trimethylates eight lysine residues in histone H1(Mujtaba et al. 2013). Histone H1 methylation driven by BaSET results in reduced activity of NF-kB response elements, silencing the inflammatory host response. It was also shown that a BaSET deletion prevents both colonization of the host by the pathogen and bacterial survival in the cellular environment.

It would be interesting to determine the function of SET-domain proteins present in other bacillus strains closely related to *B. anthracis*: *Bacillus cereus*, an opportunistic human pathogen commonly associated with food poisoning, and *Bacillus thuringiensis*, an insect pathogen (Han et al. 2006). In fact, during the last decades, thanks to the increasing number of bacterial genomes sequenced, an important number of genes putatively encoding for SET-domain containing proteins have been identified. A BLASTP search performed in 2014 recovered more that 500 bacterial genomes including SET-domain proteins (Alvarez-Venegas 2014). Many genes encoding SET-domain proteins have been identified in *B. cereus*, *Xylella fastidiosa*, *Leptospira interrogans*, *Bradyrhizobium japonicum*, or *Chlorobium tepidum* genomes and the above described family of *Chlamidyaceae*, but also in the archeal *Methanosarcina mazei* and the *Paramecium bursaria chlorella* virus-1, that encode for Gö1-SET, which methylates histone H4 *in vitro* (Manzur and Zhou 2005) and vSET that target histone H3 at lysine 27 (H3K27) (Manzur et al. 2003).

### *Legionella pneumophila*: a toolbox for protein MTases targeting the host chromatin

An excellent example among the intracellular pathogens encoding secreted SET-domain MTases, is the Gram-negative intracellular bacterium *Legionella pneumophila*, i.e. ubiquitous in aquatic environments, where it replicates within aquatic protozoa (Boamah et al. 2017, Hilbi and Buchrieser 2022). When manmade aquatic environments are contaminated with *Legionella* and susceptible humans inhale such contaminated aerosols, they may develop a pneumonic respiratory disease named Legionnaires’ disease (Mondino et al. 2020). The intracellular life cycle of *L. pneumophila* has been intensively studied. One of the major features of this thrilling pathogen is that it encodes for more than 330 effectors (Ensminger 2016). Many of these effectors, translocated in the host cell through a specialized T4SS, are so-called “eukaryotic-like proteins” or proteins with “eukaryotic-like domains” (Cazalet et al. 2004). Several phylogenetic analyses suggested that the genes encoding these eukaryotic domains have been acquired by the bacterium through horizontal gene transfer from its protozoan hosts (Gomez-Valero and Buchrieser 2013), among those, a SET-domain histone MTase named RomA (Rolando et al. 2013, Schator et al. 2021).

RomA is a secreted effector of *L. pneumophila* strain Paris, that harbors a NLS located in the N-terminal part of the protein allowing it to reach the host cell nucleus, where it specifically trimethylates lysine 14 of histone H3 (H3K14; Rolando et al. 2013). At the time, H3K14me had never been described in mammalian cells because it was likely overlooked. In fact, we now know that it is present in the human genome at very low levels, and it is activated only under specific conditions like during the stress response (Zhao et al. 2018, Zhu et al. 2021). In contrast, H3K14 is usually acetylated in mammalian cells and this mark is correlated with open chromatin and transcriptional activation (Karmodiya et al. 2012). Therefore, H3K14 methylation by RomA functions as a strong and genome-wide repressor of acetylation, leading to transcriptional decrease of gene expression during infection, in particular at promotors essential for the host cell response to infection (Rolando et al. 2013). The homologue of RomA *in L. pneumophila* strain Philadelphia, named LegAS4, also shows histone MTase activity and *L. pneumophila* strain Philadelphia and methylates H3K14 during infection (Rolando and Buchrieser 2014), but it has also been shown to interact with HP1 in the nucleolus at rRNA promotors, resulting in the activation of rRNA gene expression (Li et al. 2013).

Genome analyses of *L. pneumophila* highlighted the presence of additional protein MTases, in fact *L. pneumophila* strain Paris is predicted to also encode two protein arginine MTases (PRMTs; Cazalet et al. 2004) and a DOT1-like protein, i.e. of particular interest as it is highly conserved in the genus *Legionella* (Gomez-Valero et al. 2019). As mentioned above, DOT1, first discovered in yeast while screening for enzymes disrupting gene silencing at the telomeres (Lacoste et al. 2002), mono-, di-, or trimethylates lysine 79 on histone H3 (H3K79), which correlates with active gene transcription (Schübeler et al. 2004). So far it is the only enzyme known to methylate this mark.

Intrigued by the presence of DOT1-like proteins in all *Legionella* genomes analyzed in our previous study (Gomez-Valero et al. 2019), we conducted an in-depth phylogenetic analysis of these proteins to gain insight in their emergence within the genus *Legionella*. A first homology search in the NCBI nonredundant database confirmed that all *Legionella* genomes sequenced to date (62 different *Legionella* species) contain *dot1* genes. Whereas most of them have only one gene encoding a DOT1 protein, seven species possess several genes encoding DOT1 domains. The *Legionella fallonii* genome contains four *dot1l* genes, which is the highest number of homologues present in a *Legionella* genome.

**Figure 3.**
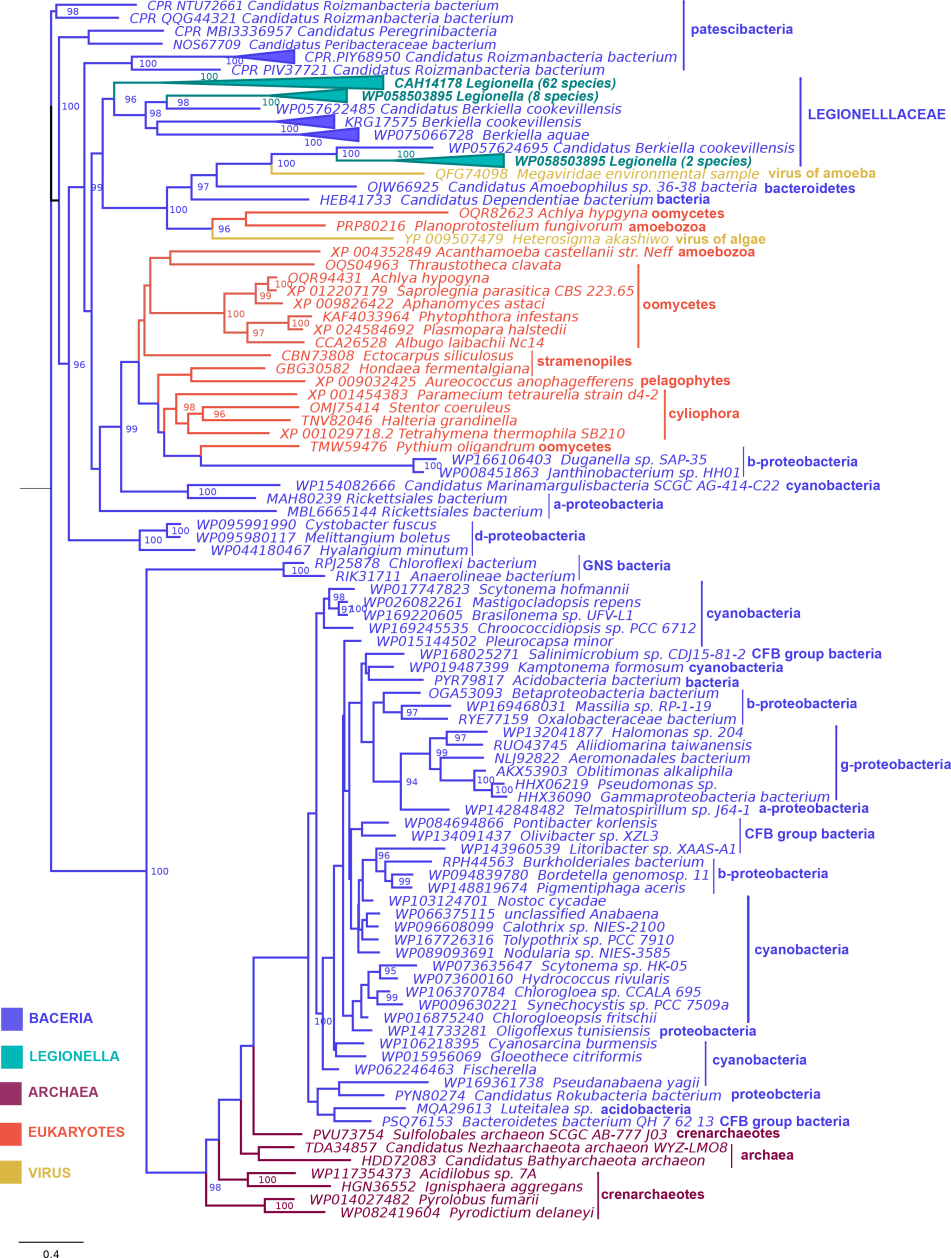
Maximum likelihood (IQ-TREE) tree of DOT1 proteins identified in *Legionella* spp. and selected DOT1 homologous sequences. Values on the right of each node correspond to ultrafast bootstrap support values (only values above 95% are shown; Minh et al. 2020). The tree has been rooted in the midpoint. The horizontal bar provides the scale for the branch length. The three different clades of *Legionella* DOT1 proteins are highlighted in green.

To analyze the origin of the *Legionella* DOT1 protein coding genes, we undertook a phylogenetic analysis including their corresponding homologous in other organisms. Homologous proteins were recruited by Blastp using as seed the *L. pneumophila* strain Paris DOT1 protein with the following filtering parameters: at least 25% of identity, a minimum coverage of 50% and an e-value of 1e^−5^ or higher. Redundant sequences (with more than 90% of identity among them) from non-*Legionella* species and outliers were removed using the multiple sequence alignment package T-Coffee (Notredame et al. 2000). The remaining sequences were aligned using M-Coffee (Wallace et al. 2006) and ambiguous positions were removed after applying an evaluation method from T-Coffee. A final reliable sequence alignment of 164 amino acids in length was obtained and used for a likelihood phylogenetic reconstruction using IQ-TREE 2 (Minh et al. 2020; Fig. 3).

In this tree, the *Legionella* DOT1 proteins are distributed in three different clades: clade one contains the 62 *Legionella* genomes analyzed, as all code for an orthologue of the *L. pneumophila* Paris DOT1 protein; clade two contains eight *Legionella* species that encode an additional DOT1 homologue; and clade three contains two *Legionella* species that contain a third DOT1 protein (Fig. 3—green). This division of *Legionella* DOT1 proteins in three different clades suggests independent origins for each of these DOT1 orthologous groups. The presence of DOT1 of clade one in all the species indicates that this protein was already present in the common ancestor of *Legionella*. However, DOT1 proteins from the other two clades are present in very few species that are not even very closely related, suggesting a more recent emergence of these proteins in the genus *Legionella*, probably through different events of acquisition.

More interestingly, when analyzing the entire obtained phylogeny, we observed that all acquired DOT1 homologues are distributed in two main clades: one containing only bacterial and archaeal sequences and another one containing the three *Legionella* clades mentioned above, clustering with homologues from eukaryotes, viruses, and bacteria (Fig. 3). Despite the heterogenicity of the organisms clustering in this second group, all of them inhabit the aquatic environment or are amoeba-related organisms. For example, the organisms closest to *Legionella* in the tree, *Berkiella* spp., also belonging to the order Legionellaceae, are intracellular bacteria of freshwater amoeba. Similarly, in the same clade we find the amoeba endosymbiont *Candidatus amoebophilus*, the amoeba virus *Megaviridae*, or bacteria typically isolated from amoeba such as *Candidatus dependentiae*. Other bacteria in the same cluster such as *Roizmanbacteria* spp or *Duganella* are also prevalent in aquatic environments. In the same clade, there are also eukaryotic sequences that are all from protists belonging to Amoebozoa and the eukaryotic clade SAR. This clade includes Stramenopiles, Alveolates, and Rhizaria and contains a large diversity of lineages including amoebae, ciliates, and flagellates that live almost everywhere (Grattepanche et al. 2018). Among them there are ciliates and amoeabae such as the well-established natural host of *Legionella*, *Acanthamoeba castellanii*.

Taken together, these analyses strongly suggest that the horizontal transfer of genes encoding proteins with DOT1 domains has taken place many times between prokaryotes, eukaryotes, and viruses cohabiting in the same niche and support the hypothesis of a selective pressure to acquire and maintain the *dot1 L* gene for amoeba-related organisms such as *Legionella* spp. Our results might also indicate that DOT1 is important to create a niche inside eukaryotes probably by modifying histones, as it was shown for RomA.

## Perspectives

Efficient high-resolution mapping of bacterial DNA-methylation events has only recently become possible with the advent of single-molecule real time sequencing—SMRT (Zhao et al. 2020). The mapping and characterization of bacterial DNA methylomes by SMRT, or potentially new future technologies, will allow to discover new MTases, but also novel genes epigenetically regulated by DNA methylation. This is important, as epigenetic changes may provide an early marker of potential alterations in gene expression leading to disease. In addition, genetic repertoires stably expressed during infection would be identified by the characterization of phasevarions (Seib et al. 2020).

In the context of infection by bacterial pathogens, the identification of novel DNA MTase-controlled genes, that could be associated to virulence traits, will allow to characterize, and potentially repress, medically relevant biological processes by designing new therapeutic approaches. Thus, bacterial MTases may emerge as promising targets for the development of novel epigenetic inhibitors (Ceccaldi et al. 2013), in particular in the context of antibiotic resistance. Indeed, bacteria use methylated nucleobases to resist antibiotics, and DNA MTase inhibitors may be used to enhance the therapeutic activity of antibiotics. It has been shown, e.g. for *E. coli* that DNA adenine MTase deficiency potentiates the lethal action of antibiotics by increasing the bacterial activity of beta-lactams and quinolones (Cohen et al. 2016). Also, rRNA methylation is involved in antibiotic resistance. Most known inhibitors of protein synthesis bind to the functional sites of ribosomes, and when methyl groups are introduced, they can spatially overlap with the antibiotic binding site (Osterman et al. 2020).

Furthermore, MTases may also emerge as promising targets for the identification and the development of new vaccines against human-adapted pathogens (Phillips et al. 2019b). For example, live vaccines against *Salmonella* spp. and *Yersinia pseudotuberculosis*, have been developed using strains that carry MTase mutations (Heithoff et al. 2015) (Taylor et al. 2005).

Several MTases have been described to play a role as nucleomodulins during bacterial infection, and many genes putatively encoding MTases remain to be characterized during infection. Furthermore, some bacterial MTases have been identified to modify the transcriptional host cell response by targeting nonhistone proteins. One example is the MTTL20 homologue encoded by *Agrobacterium tumefaciens*, a specific protein–lysine MTase that targets ribosomal protein L7/L12 and the β-subunit of electron transfer flavoprotein (ETF-β; Falnes et al. 2016). Another example is enteropathogenic *E. coli* (EPEC), a bacterium classified as a major diarrheagenic agent, transmitted by contaminated water or undercooked food, and identified as one of the major causes of mortality in children under five. EPEC *E. coli* encode for NleE, an S-adenosyl-L-methionine (AdoMet)-dependent MTase, that modifies Cys673 in the Npl4 zinc finger (NZF) domain of the host signaling adaptor protein TAB2, and Cys692 in the same portion of the protein TAB3, both involved in the signaling via Toll-like or TNF receptors, and IL-1 (Zhang et al. 2016).

In contrast, RNA MTases have not been classified as nucleomodulins to date. Although recent studies showed the presence of m^6^A modification on viral and cellular RNAs during infection (McFadden and Horner 2020), very little is known about bacteria (directly) modulating m^6^A of eukaryotic mRNA. However, bacterial infection increases m^6^A levels in nascent transcripts, in particular in transcripts related to histone modifications (Wu et al. 2020), and m^6^A may be involved in regulating the response to LPS, as both m^6^A reader and writer proteins (YTHDF2 and METTL3) have been shown to play a role in regulating the cell response to bacteria and cytokine production (Yu et al. 2019) (Feng et al. 2018). Thus, bacterial RNA MTases may also prove as important virulence factors and perhaps as targets for enhancing treatment of bacterial infections in the future.
